# Comparison of growth and physiological characteristics between roughstalk bluegrass and tall fescue in response to simulated waterlogging

**DOI:** 10.1371/journal.pone.0182035

**Published:** 2017-07-27

**Authors:** Mingyang Liu, Andrew Hulting, Carol Mallory-Smith

**Affiliations:** Department of Crop and Soil Science, Oregon State University, Corvallis, Oregon, United States of America; University of Illinois at Urbana-Champaign, UNITED STATES

## Abstract

Roughstalk bluegrass (*Poa trivialis*) is a weed in cool season grass seed production fields in Oregon. Populations of this weed are often greater in fields prone to waterlogging. A greenhouse study was conducted to investigate the morphological and physiological differences between recently established roughstalk bluegrass and tall fescue (*Lolium arundinaceum*) plants in response to simulated waterlogging. Differences in root morphological development and root respiration were found between waterlogged tall fescue and roughstalk bluegrass. Plants after 4 weeks of waterlogging, leaf number, plant height, and root biomass were reduced more in tall fescue than in roughstalk bluegrass plants. The root length increased 6% in waterlogged tall fescue plants, and decreased 42% in waterlogged roughstalk bluegrass plants, which lead to a shallower root system in roughstalk bluegrass. Root aerenchyma area increased more in waterlogged roughstalk bluegrass than in tall fescue. Alcohol dehydrogenase and lactate dehydrogenase activities increased in the roots of both species, but not in the leaves. The increases were greater in tall fescue than in roughstalk bluegrass. Turf quality, aboveground biomass, photosynthetic capacity, and water-soluble carbohydrate concentrations were reduced by waterlogging, but there were no differences over time or species. Thus, the shallower root system, larger aerenchyma, and reduced fermentation rates were the characteristics most likely to contribute to better waterlogging tolerance in roughstalk bluegrass compared to tall fescue and invasion of roughstalk bluegrass in waterlogged cool season grass seed fields.

## Introduction

Roughstalk bluegrass (RB) is a perennial cool season grass species, which also is a weed in cool-season home lawns and golf courses. In grass seed crops, RB can be competitive and contaminate the harvested grass seed [1]. The small size (5.5 million per kilogram) [2] and sticky nature of RB seed increase the difficulty and cost of seed cleaning and conditioning. There are very limited control methods, either chemical or cultural, for this weed species in mixed grass stands. In the past decade, complaints by growers about RB as a weed problem have increased in the Willamette Valley of Oregon, where nearly two-thirds of the cool-season grass seed in the United States is produced [3]. The invasion of RB in cool season grass seed crops, such as tall fescue (TF) and perennial ryegrass (*Lolium perenne*), often occurs in flooded soils. RB appears to be more tolerant to the stresses caused by waterlogging. The number of studies on RB and other grasses such as TF in response to waterlogging are limited.

Soil flooding is one of the major abiotic problems in some lowland crop production areas [4]. Anoxia (anaerobic) or hypoxia (mixed anaerobic and aerobic) conditions may be found in flooded soils because air pores in saturated soils are filled by water, and oxygen diffusion is blocked. Soil redox potential (Eh), which is a measure of electrochemical potential or electron availability within the soil, often is used to quantify the level of waterlogging in soil. Lower soil Eh indicates reduced oxygen and longer or increased flooding in the soil.

Morphological adaptation can help plants mitigate oxygen deprivation during waterlogging or submergence [5]. Waterlogging may result in existing root systems being replaced with new, morphologically distinct adventitious or lateral root systems [6]. Because oxygen concentration in the surface water or upper soil layers is greater than in deeper soil layers, adventitious and lateral roots can absorb oxygen more effectively from these areas. The alternative root systems have been observed in both dryland species (*e*.*g*. pea, *Pisum sativum*) and marsh plants (*e*.*g*. *Melaleuca* spp.) under waterlogging stress [7, 8, 9]. In addition to the changes in roots, morphological changes of aboveground parts influence waterlogging tolerance in some species. A study conducted by Raskin and Kende [10] indicated that the elongation rate of submerged deepwater rice (*Oryza sativa*) shoots was faster than normal, which reduced damage to the rice seedlings.

Aerenchyma is a structure that forms in the leaves, stems and roots of some plants, which enhances internal aeration between or within shoots and roots [11, 12]. This structure increases the internal gas filled space, and therefore improves the gas diffusion efficiency between the environment and the endodermis [13, 14]. A study conducted on rice roots indicated that some well-connected channels in a longitudinal direction along the root axis were formed by the remnants of lysed cells, which provided extra gas pathways for internal root cells [15]. Because the roots with aerenchyma have fewer cells in the same volume due to the programmed cell death, these roots consume less oxygen in comparison with the roots without aerenchyma [16].

Metabolic responses to oxygen deficiency are essential for plant survival under waterlogging conditions, especially for root cells [17, 18]. One of the key processes for oxygen deficiency tolerance is to maintain energy production, usually via modified anaerobic carbohydrate catabolism [16, 18]. Plant fermentation rates can either slow down or accelerate in response to a quickly reduced oxygen concentration [19]. Germinating lettuce (*Lactuca sativa*) seeds appeared to slow down anaerobic carbohydrate catabolism by 65% to survive short term anoxia [20]. In contrast, accelerated fermentation under oxygen deficiency has been observed in many species, for example in submerged rice coleoptiles [21, 22]. One benefit of an increased fermentation rate is maintenance of energy production needed to support plant activity. However, an increased fermentation rate does not always contribute to oxygen deficiency tolerance. Pea root tips may only survive anoxia for a very short time, even with an increased fermentation rate [16, 19]. Problems caused by increased fermentation rates include accumulation of toxic anaerobic metabolites and lack of respiration substrates [16]. The toxic metabolite may cause cell injury and death, while a greater consumption rate of respiration substrates may result in substrate deficiency, eventually reducing energy production before oxygen concentration recovery [16, 23]. Thus, the regulation of the fermentation rate is important for waterlogging tolerance [24, 25]. Alcohol dehydrogenases (ADHs) and lactate dehydrogenases (LDHs) are two important fermentation regulators often used to quantify the rates of ethanol fermentation [16, 26]. ADHs in grass species generally increase under oxygen deficiency conditions [27, 28], but in RB ADH activity was found to be less sensitive to waterlogging than in other grass species [29]. LDHs are another important enzyme family found in animals, plants, and prokaryotes, which regulates the lactate pathway by catalyzing the conversion between pyruvate and lactate [16, 30]. LDH activity often is used as a supplemental parameter to measure fermentation activities of higher plants [31, 32].

Water soluble carbohydrates (WSC) are the primary fermentation substrates in higher plants [33, 34]. WSC are the most essential components of plant nutrition translocated from a carbohydrate source (e.g. leaves, stems) to a carbohydrate sink (e.g. roots, fruits) [35, 36]. WSC reserves may be reduced in a waterlogged plant, because of the altered balance between photosynthesis and carbohydrate metabolism [37]. Waterlogging induced WSC changes are regarded as one of the relevant factors that influenced the fermentation rate in some grass species [5, 38, 39].

Because photosynthesis provides plants with energy and carbohydrates, photosynthetic adaption is regarded as a major component of waterlogging tolerance [40]. Photosynthetic light-response curves describing the photosynthetic capacity, efficiency, and other parameters are commonly used to evaluate photosynthesis performance under environmental stress [41]. Waterlogging treatments reduced chlorophyll content in some grass species [42, 43]. Although high water potential induced stomata closure is considered to be the major reason for reduced photosynthesis during short-term flooding [44], chlorophyll content reduction may eventually result in reduced photosynthetic capacity during long-term waterlogging stress [43, 45, 46].

Invasion of some non-native species in wetlands was successful because of increased oxygen deficiency tolerance or photosynthesis during short-term flooding [40, 47]. Studies are rare in waterlogged agricultural systems. However, it is possible that waterlogging provides an advantage for RB in grass seed crop fields due to differences in waterlogging tolerance among these species.

Tall fescue is an important grass seed crop in the Willamette Valley, and is one of the crops that is often invaded by RB. Tall fescue is relatively more tolerant to waterlogging than some other turf grasses species such as Kentucky bluegrass (*Poa pratensis*) [48, 49]. This study was conducted to compare the relative waterlogging tolerance between RB and tall fescue. The specific objectives of this study were to evaluate the waterlogging influences on the morphology, metabolism, and photosynthesis of these two grass species.

## Materials and methods

### Plant material and general growing conditions

**Fig 1:**
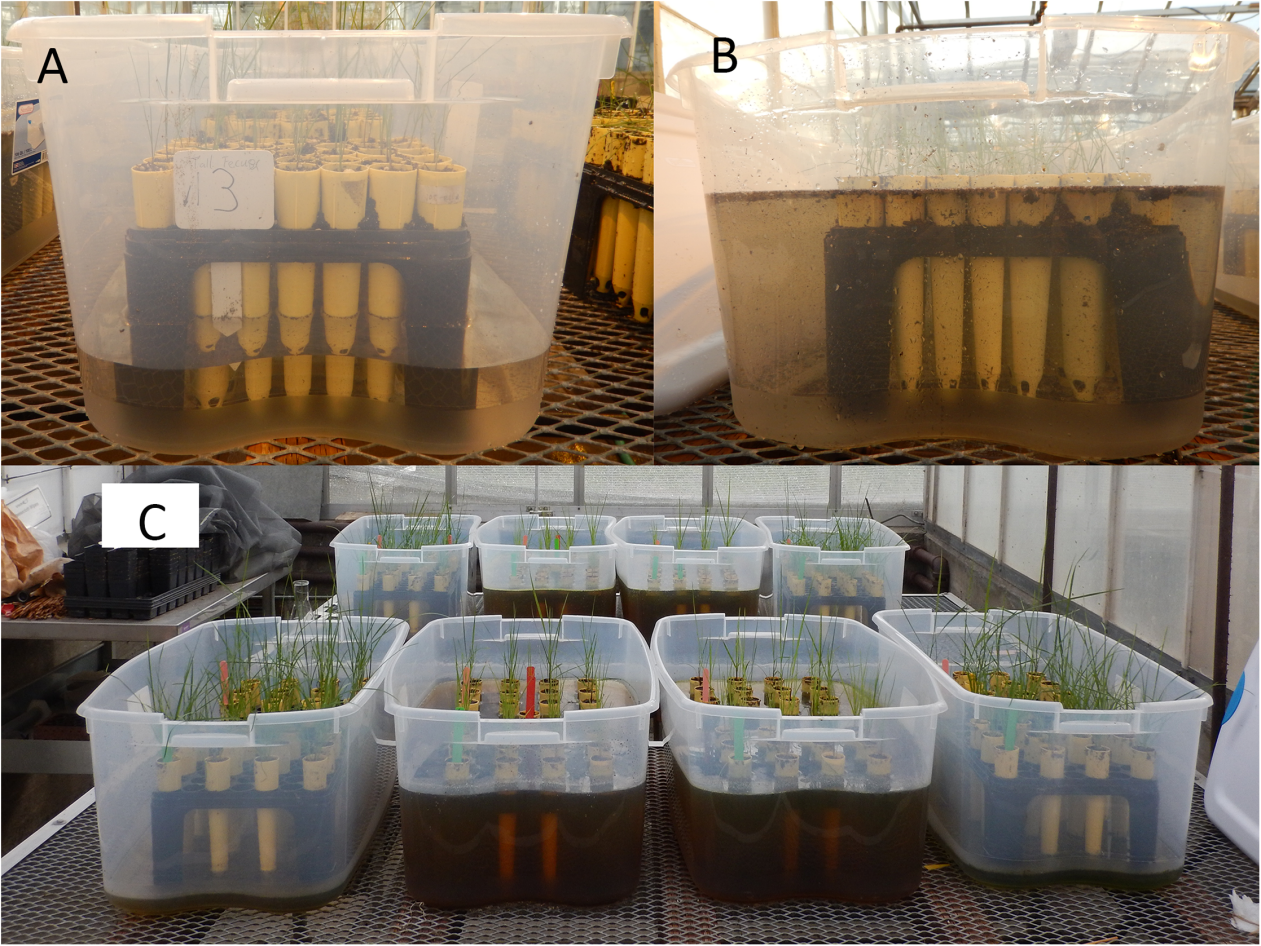
Experimental setup in the greenhouse. (A) Control; (B) Waterlogging treatment; (C) Four waterlogging treatments and four controls were arranged in a randomized complete block design in the greenhouse study.

A commercial RB cultivar (“Quasar”, Seed Research of Oregon, Tangent, OR) and a TF cultivar (“Rebel XLR”, Pennington, Madison, GA) were used in this study. Studies were conducted in the greenhouse and laboratory at Oregon State University, Corvallis, OR. The greenhouse environment was 25 /20°C day/night with ambient sunlight plus grow lights providing 14 h light above 25 mW cm^−2^ per day. Seeds were germinated in petri dishes in a germination chamber. When the coleoptiles reached 1.5 cm, a seedling was planted in a 21 cm × 4.5 cm pot filled with potting soil (Sunshine Mix 1 Potting Mix; Sun Gro Horticulture, Bellevue, WA) and placed in 32 × 58 × 12 cm clear plastic containers (Fig 1), when leaf numbers of both species ranged from 3 to 5 with most the plants having 4 leaves. The waterlogging treatment was applied on the same day the pots were placed in the plastic containers. For the waterlogging treatment, the plastic containers were filled with water to the soil surface. In the control containers, the water level was kept at 18 cm below the soil surface. The water level was checked daily and water was added as necessary. Treatments were applied continuously for four weeks. The study was a randomized complete block design with two treatments, waterlogged and control, and four replications. The study was repeated in time.

### Soil redox potential

Rhizosphere redox potential, Eh, was measured with an oxidation-reduction potential probe (WD-35649-50, Oakton Instruments, Vernon Hills, Il 60061). The probe was inserted to a 15 cm depth. The probe was connected to and read using a benchtop pH meter (Accumet Research AR50, Fisher Scientific, Waltham, MA). The soil Eh was quantified at 1, 2, 3, and 4 weeks after treatment. Two readings were made in each container at each timing.

### Morphometric and biomass measurements

The morphological parameters, biomass, and turf quality were measured at 1, 2, 3, and 4 weeks of treatment (WOT). For each treatment, six plants were randomly sampled from each container (24 plants per species) at each date. Turf quality was visually rated as an integral of color, shape and health on a scale from 0 (death, dry leaves) to 10 (healthy, green leaves). Plant height was measured, and leaf number were counted. Aboveground biomass and roots were harvested, washed under tap water to remove soil, and root length was measured. The harvested leaves and roots were dried for 72 hr at 60°C and weighed. Vertical distribution of root biomass was measured, and was calculated as a ratio of gram root dry biomass per centimeter root depth (g DW cm^-1^).

### Aerenchyma formation examination

Aerenchyma formation was examined at 1, 2, 3, and 4 WOT. Root samples were collected from 2 randomly selected plants in each treatment (16 plants per species). Root samples were washed with deionized water, and cut to 10 mm sections. The selected root sections were placed into fixative provided by OSU Electron Microscopy Facility (Oregon State University, Corvallis, OR 97331). After soaking in fixative for 8 to 24 hr, root samples were rinsed with 0.1 M sodium cacodylate for 10 min. The rinsing procedure was completed three times. The fixed root samples were sent to the OSU Electron Microscopy Facility for further preparation. The final prepared root samples were viewed with a field emission scanning electron microscopy (FESEM) using an FEI QUANTA 600F environmental SEM with an energy-dispersive X-ray (EDX) attachment.

**Fig 2:**
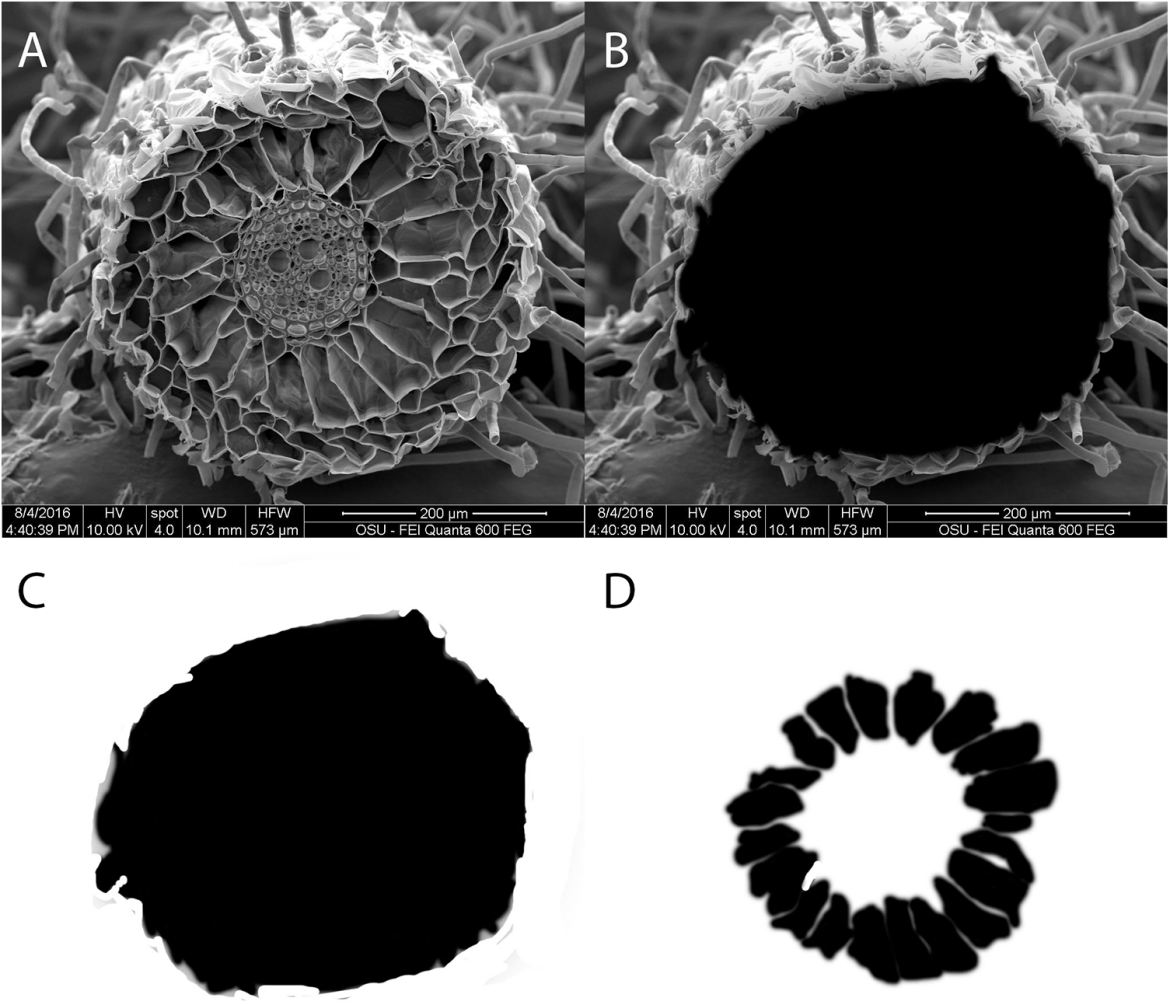
Representative images used in digital quantification of aerenchyma area. (A) Original digital image of a tall fescue root cross section; (B) root cross section covered with black ink on a new image layer; (C) total cross sectional area; (D) aerenchyma spaces. Percentage of aerenchyma (44%) was calculated by dividing the number of pixels in (C) (pixels: 104932) by the number of pixels in (D) (pixels: 46917).

The image analysis and aerenchyma area quantification methods were designed based on Maricle and Lee [50]. Digital images of root cross sections were analyzed with Photoshop CC 2014 software (Adobe, San Jose, CA 95113) to measure the percent root aerenchyma by area in the root cross section. In the digital images of root cross section (Fig 2A), a new layer in Photoshop CC 2014 was created to cover the original image. On this new layer, painting tools were used to trace the root cross section area in black ink (Fig 2B). The area of the root cross section was quantified by reading the pixels of the new layer, after withdrawing the original image (Fig 2C). The area of the aerenchyma was quantified using the same method (Fig 2D). The percent aerenchyma in the cross section was calculated by dividing the number of pixels in aerenchyma area by the number of pixels in the cross-section area. Images of aerenchyma of each replication were taken from three randomly selected cross sections 2 to 10 cm from the root tip, and the data were averaged (Fig 2).

### Metabolic responses to waterlogging

Metabolic responses of RB and TF to waterlogging were evaluated by measuring WSC content, ADH, and LDH activities after 1, 2, 3, and 4 weeks of treatment. Crude protein was extracted following the methods described by Proels and Huckelhoven [51]. For each replication, samples were randomly harvested from fresh leaves or roots of four plants per species. A total of 16 plants were harvested per species per treatment. Harvested plant tissue was ground in a mortar with liquid nitrogen. One hundred and fifty mg of the ground sample was extracted with 1.5 ml solvent which contained 50 mM Tris buffer, 5 mM MgCl_2_, 10 mM sodium borate, 1mM ethylenediaminetetraacetic acid, 1 mM phenylmethylsulphonyl fluoride, and 5mM dithiothreitol. Extract was purified by centrifuging at 13,000 g at 4°C for 10 min. The protein concentration was quantified using a Bio-Rad Quick Start^TM^ Bradford protein assay kit (Bio-Rad Laboratories, Hercules, CA) following the instruction manual.

ADH activity was determined based on the method described by Proels and Huckelhoven [51]. A 1.5 ml reaction mix containing 0.65 ml 50 mM pH 8.0 sodium phosphate buffer, 0.05 ml 95% (v/v) ethanol, 0.75 ml 15 mM β-NAD solution, was added to 0.05 ml crude protein sample solution and mixed in a 2 ml centrifuge tube. After the mixture was vibrated and incubated at 25°C for 5 min, the photon absorbance of the sample solution was measured using a multi-titer spectrophotometer (Versa MAX microplate reader with Soft MAX Pro, Molecular Devices) for 15 min. The ADH activity was determined by measuring the increased rate of photon absorbance at 340 nm which resulted from reduction of β-NAD. The specific ADH activity was calculated as units of ADH activity per mg FW crude protein (U/mg FW). One unit of ADH activity was defined as the amount of ADH that converted 1.0 nmole of β-NAD to β-NADH per minute at pH 8.0 and 25°C. The activity of LDH was determined based on the method described by Wang et al. [26]. A 1.5 ml reaction mixture containing 65 μl of extracted crude protein sample, 100 mM Tris–HCl (pH 8.0), 30 μM 4-bromopyrazole, 0.18 mM β-NADH, plus 3.0 mM sodium pyruvate were mixed in a 2 ml centrifuge tube. The photon absorbance of the sample solution was measured immediately after the mixture was vibrated. The LDH activity was determined by using a multi-titer spectrophotometer to measure the decrease of absorbance at 340 nm resulting from the oxidation of β-NADH for 15 min. The specific LDH activity was calculated as units per mg crude protein (U/mg). One unit LDH activity was defined as the amount of LDH that oxidized 1 nmole β-NADH per minute at 25°C and pH 7.3.

The WSC extraction was based on the method described by Buysse and Merckx [52]. Leaf and root samples were harvested from four plants per treatment per species (32 plants of each species) at each date. Samples were dried at 60°C for 72 hr and ground in a mortar with liquid nitrogen. Ten mg ground leaf or root tissue were placed in a 2 ml centrifuge tube containing 1.5 ml deionized water and boiled for 10 min. The tube was centrifuged at 13,000 g for 10 min and the supernatant collected. The extraction procedure was repeated, and the supernatants combined for analysis.

The extracted WSC concentration was quantified using the anthrone method described by Yemm and Willis [53]. Anthrone reagent was made by dissolving 1 g anthrone in 500 ml of 72% sulphuric acid. One ml WSC extract and 5 ml ice-cold anthrone reagent were mixed in a 10 ml test tube. The mixture was heated for 11 min in a 100°C water bath and cooled on ice to 0°C. Photon absorbance of the mixture was read at 630 nm with a multi-titer spectrophotometer. Concentration was determined by comparison to a standard curve.

### Photosynthesis and chlorophyll measurements

Photosynthetic response to waterlogging was evaluated via a light response curve using a portable photosynthesis system (LI-6400XT, Li-Cor, Inc., Lincoln, NE, 68504). Net photosynthetic rates (A, μmol CO_2_ m^−2^ s^−1^) were quantified using system software (OPEN versions 6.2). At 1, 2, 3, and 4 weeks of treatment, measurements were conducted between 10:00 and 15:00, when solar radiation was at maximum intensity. One plant of each species per replication was selected for the light response curve measurement. Two to three fully expanded leaves from each plant were placed side by side with no overlap into the leaf chamber. The leaf area in the chamber was determined with a portable laser area meter (CI-202, CID Bio-Science, Camas, WA, 98607), and the photosynthetic data were adjusted using the corrected area. Light response curves were generated with a built in function of the system with seven radiant intensity levels of 1500, 1000, 500, 300, 150, 50, and 0 umol m^-2^ s^-1^. The relative humidity in the chamber was 25% at the chamber temperature of 25°C. The CO_2_ concentration in the chamber was 360 ppm. Seven to 15 min were required for both photosynthesis and stomatal conductance to stabilize.

Following the photosynthetic measurement, fresh leaves were harvested for chlorophyll content estimation. Chlorophyll was estimated based on the method described by Vernon [54]. Four plants of each species were randomly selected from each treatment replication (32 plants of each species). Approximately 100 mg (Wt1) of deveined leaf tissue from each plant were ground in a mortar with liquid nitrogen to a fine paste in 1 ml 80% chilled acetone, and the suspension centrifuged at 13,000 g for 15 min. Absorbance (A) of the supernatant of each species was measured at 645 and 663 nm in a multi-titer spectrophotometer. Extinction coefficients were 45.6 and 9.27 Lg^-1^ cm^-1^ at 645 and 663 nm, respectively [55]. Chlorophyll content was calculated using formula (1):

$$total\;chlorophyll\;content\;in\;fresh\;leaf\;tissue\;(mg/g\;FW)=\frac{A663\times 8.02+A645\times 20.2}{0.3\times Wt1}$$ (1)

### Data analysis

Open source statistical software R (R Development Team, http://www.r-project.org/) was used to analyze the effects of the waterlogging treatment. ANOVA tests were performed to analyze the responses between RB and TF to waterlogging. Means were separated based on Duncan’s multiple range test with P-value less than 0.05.

## Results

ANOVA analysis indicated that the data within the same treatment were not different among replications, thus, data for the same treatment were combined for analysis.

### Soil redox potential

The simulated waterlogging treatment created a lower redox potential (Eh) in soils. The average soil Eh was reduced by 20, 36, 34 and 35% by waterlogging after 1, 2, 3 and 4 WOT, respectively (Table 1).

**Table 1:** Soil redox potential, (Eh), readings during experiment.

Duration	Eh (mV)^a^	
	cnt^b^	wl
1 week	341	272
2 week	330	212
3 week	388	257
4 week	353	228

^a^mV = millivolts

^b^cnt = control; wl = waterlogging treatment

### Morphometric and biomass measurements

All of the plants of RB and TF survived the 4-week waterlogging treatment. However, all the measured parameters including turf quality, leaf number, plant height, aboveground dry biomass, root dry biomass, root length, and root distribution in both RB and TF were influenced by waterlogging.

Turf quality and aboveground dry biomass were reduced by waterlogging (Table 2), but there were no differences between the species. At the end of the study (4 WOT), turf quality of treated TF and RB plants was 54% and 57% of the untreated control, respectively. The major symptoms of waterlogging damage included yellow and wilting leaves. Aboveground dry biomass of waterlogged TF and RB plants was 76 and 81% of the untreated control, respectively. The treatment reduced leaf number, plant height, and root dry biomass of TF more than of RB. Leaf number, plant height, root dry biomass, root length, and root distribution in the waterlogging treatment were 68, 58, 57, 106, and 54% of the untreated control for TF, and 70, 65, 87, 58, and 150% of the untreated control for RB (Table 2). Waterlogging reduced root length in RB (58%), but promoted root length in TF (106%). There was no significant waterlogging damage observed in the roots of either species. Based on visual observations, the roots of RB were finer but were greater in number compared to TF.

**Table 2:** Turf quality, leaf number, height, aboveground dry biomass, root dry biomass, root length, and root distribution of tall fescue (TF) and roughstalk bluegrass (RB) under control and waterlogging conditions.

Species	Treatment^a^	TQ^b^	LN	H	ADB	RDB	RL	RD
				cm	g	g	cm	g dw cm^-1^
		Week 1^c^						
TF	cnt	9.72a	10.00a	26.09a	0.61a	0.68a	14.06a	0.048a
	wl	8.72b	10.41a	18.28b	0.46ab	0.53b	18.75b	0.021b
RB	cnt	9.88a	18.78b	22.03c	0.36b	0.81c	19.88c	0.041c
	wl	8.69b	15.28c	14.28d	0.36b	0.58d	12.56d	0.046ac
		Week 2						
TF	cnt	9.69a	12.25a	29.72a	0.70a	0.68a	16.94a	0.040a
	wl	5.00b	8.88b	21.63b	0.50b	0.58a	27.31b	0.021b
RB	cny	9.84a	18.53c	24.38c	0.39c	0.87b	20.50c	0.043a
	wl	6.09b	15.47d	16.91d	0.35c	0.49c	13.56d	0.036c
		Week 3						
TF	cnt	9.25a	9.44a	33.00a	1.00a	1.02a	44.34a	0.023a
	wl	4.84b	8.34b	17.66b	0.93a	0.76b	41.34b	0.018a
RB	cnt	9.56a	31.06c	26.38c	0.53b	0.91ab	23.66c	0.038b
	wl	5.38b	20.97d	16.50d	0.46c	0.50c	19.91d	0.025c
		Week 4						
TF	cnt	9.25a	12.78a	37.47a	1.34a	1.53a	38.56a	0.040a
	wl	5.03b	8.66b	21.72b	1.02b	0.87b	40.78b	0.021b
RB	cnt	9.13a	28.81c	27.41c	0.64c	0.77bc	31.16c	0.025bc
	wl	5.22b	20.06d	17.84d	0.52c	0.67c	18.16d	0.037a

^a^ cnt = control; wl = waterlogging treatment.

^b^ TQ = turf quality; LN = leaf number; H = plant height; ADB = aboveground dry biomass; RDB = root dry biomass; RL = root length; RD = root distribution.

^c^ data represent means of 48 individuals. Means within the same week followed by the same letter in the column are not different based on Duncan’s multiple range test at 0.05 probability.

### Aerenchyma formation examination

**Fig 3:**
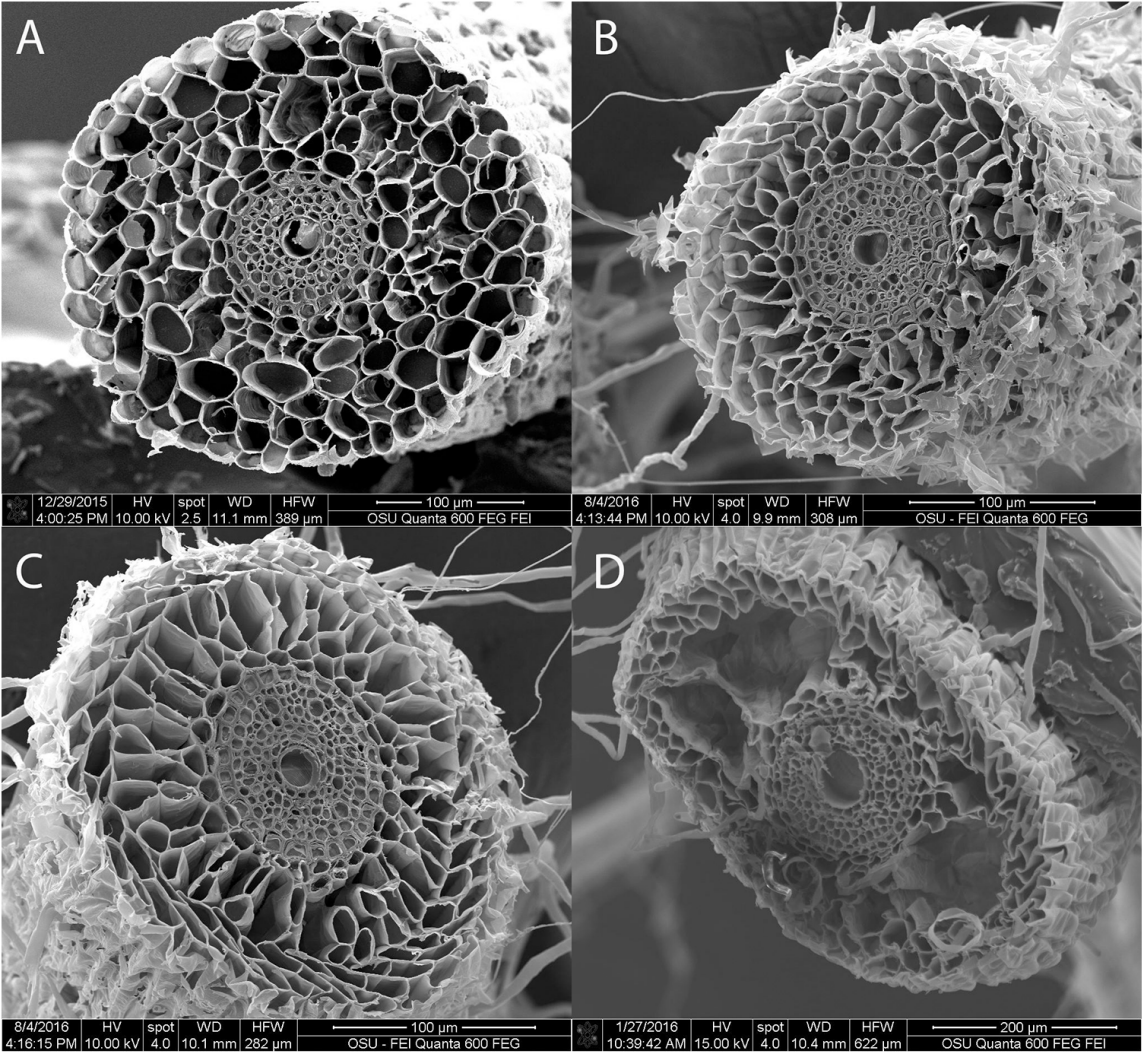
Images of root cross sections in roughstalk bluegrass (RB). Examples of root cross sections with 0 (A, control), 18 (B, control), 34 (C, waterlogged), and 51% (D, waterlogged) aerenchyma by area. Aerenchyma occurred in both control and waterlogged plant roots.

**Fig 4:**
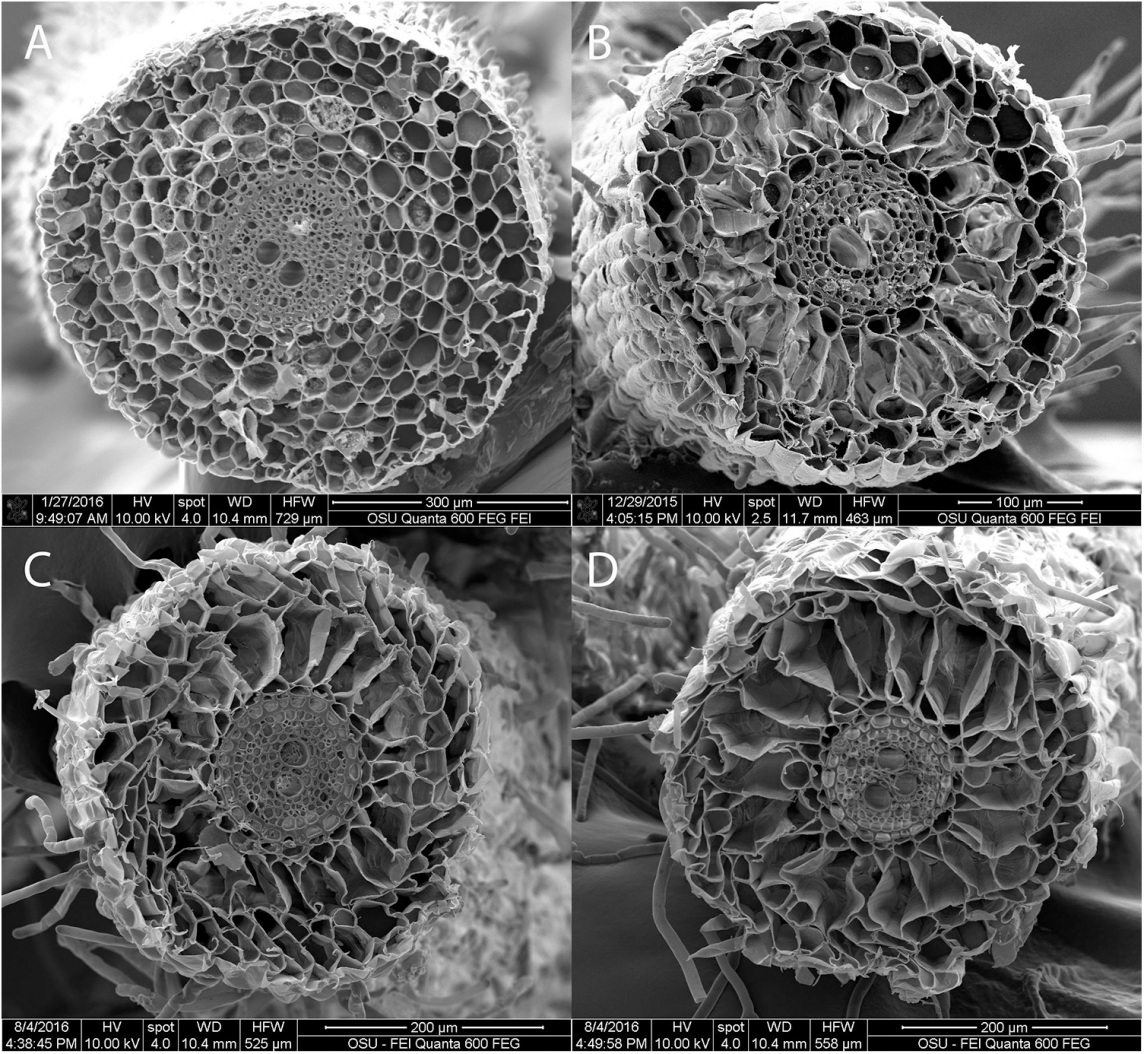
Images of root cross sections in tall fescue (TF). Examples of root cross sections with 0 (A, control), 24 (B, control), 29 (C, waterlogged), and 48% (D, waterlogged) aerenchyma by area. Aerenchyma occurred in both control and waterlogged plant roots.

Lysigenous type aerenchyma was observed in the root cortex of both RB and TF, and in both control and waterlogged plants (Figs 3 and 4). The average aerenchyma area in root cross sections ranged from 14 to 34% in TF, and from 13 to 38% in RB (Table 3). The waterlogging induced aerenchyma increases were greater in RB than TF. Aerenchyma areas increased in waterlogged TF at 1, 2, 3, and 4 WOT. In waterlogged RB roots, aerenchyma areas increased at 2, 3, and 4 WOT. At the end of the study, the aerenchyma was 23 and 34% in the control and waterlogged TF roots, and 29 and 38% in the control and waterlogged RB roots, respectively.

**Table 3:** Changes in aerenchyma area in the root cross sections of tall fescue (TF) and roughstalk bluegrass (RB) during the 4 week greenhouse study.

Population	Treatment^a^	Aerenchyma area^b^ (%)			
		Week 1	Week 2	Week 3	Week 4
TF	cnt	14aA	23bA	24bA	23bA
	wl	22aB	28bB	31bcB	34cC
RB	cnt	13aA	21bA	28cA	29cB
	wl	16aA	28bB	34cB	38cC

^a^ cnt = control; wl = waterlogging treatment.

^b^ Data as the percentage of aerenchyma by area in root cross section. Data represent the mean of 8 replications. Means followed by the same lower-case letter in the row and upper case letter in the column are not different based on Duncan’s multiple range test at 0.05 probability.

### Metabolic responses to waterlogging

**Fig 5:**
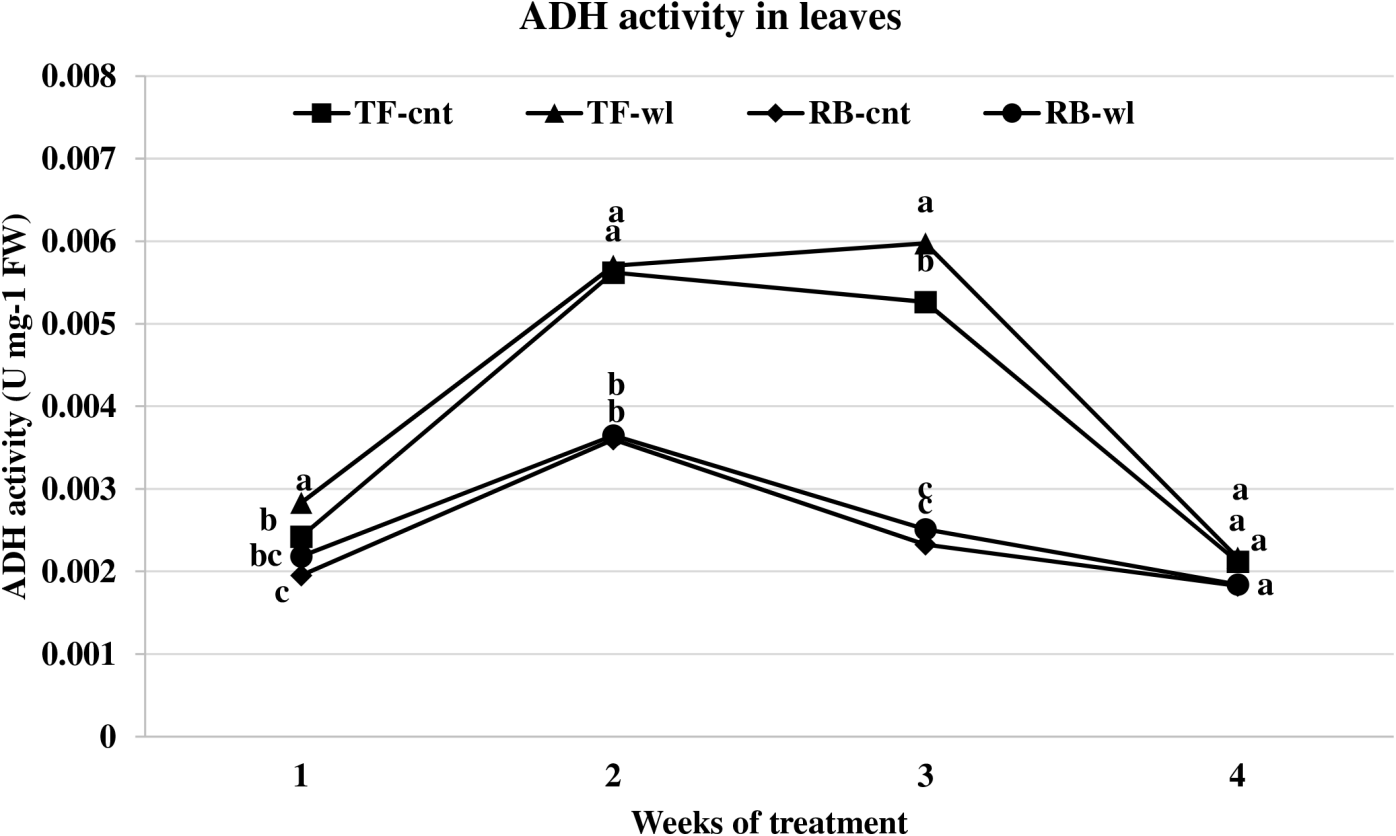
ADH activity changes in leaves of tall fescue (TF) and roughstalk bluegrass (RB). ADH activity changes in leaves of control tall fescue (TF-cnt), control roughstalk bluegrass (RB-cnt), waterlogged tall fescue (TF-wl), and waterlogged roughstalk bluegrass (RB-wl) during the 4-week greenhouse study. Data represent means of 32 individuals. Means in the same week with the same letter are not different based on Duncan’s multiple range test at 0.05 probability.

**Fig 6:**
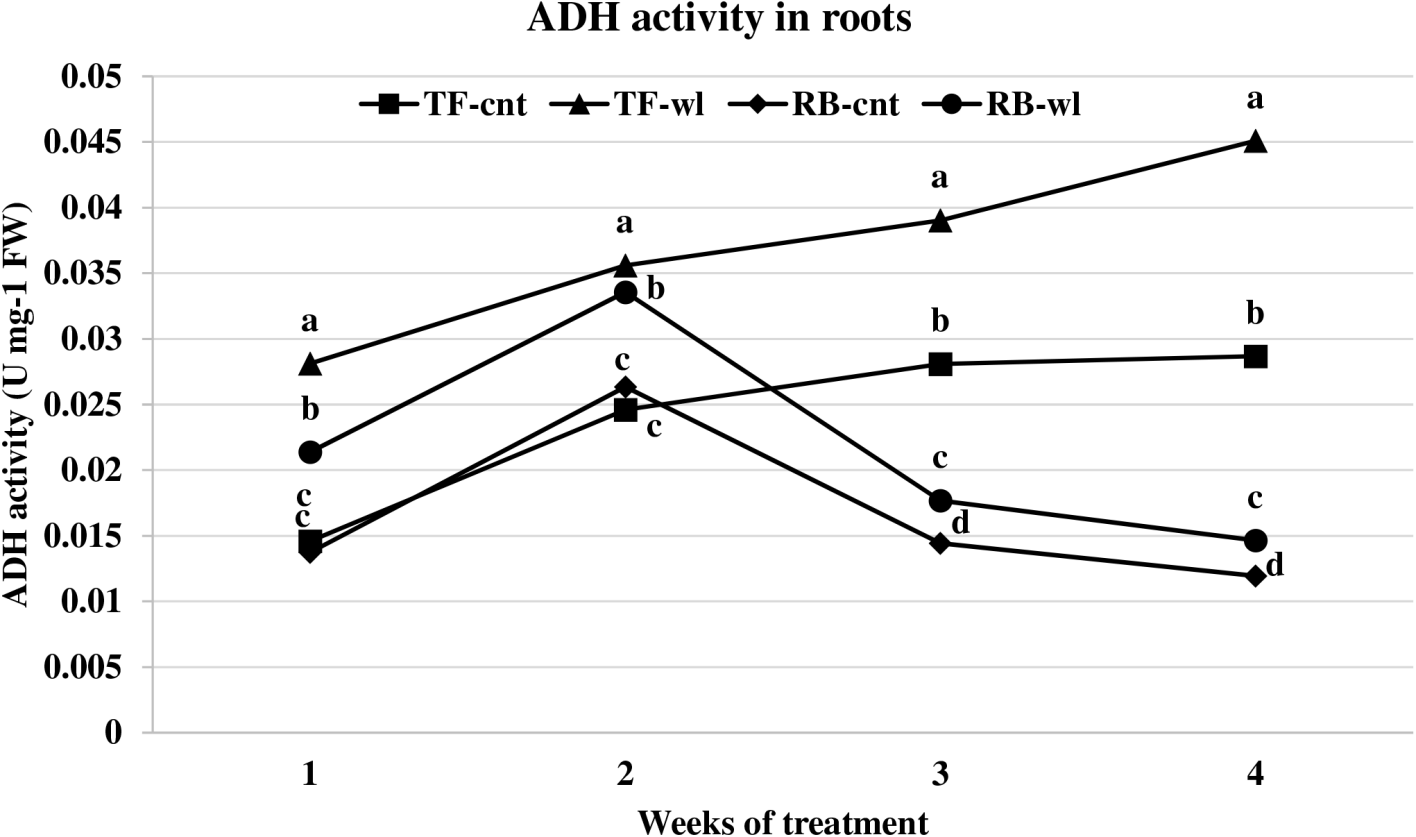
ADH activity changes in roots of tall fescue (TF) and roughstalk bluegrass (RB). ADH activity changes in roots of control tall fescue (TF-cnt), control roughstalk bluegrass (RB-cnt), waterlogged tall fescue (TF-wl), and waterlogged roughstalk bluegrass (RB-wl) during the 4-week greenhouse study. Data represent means of 32 individuals. Means in the same week with the same letter are not different based on Duncan’s multiple range test at 0.05 probability.

Waterlogging induced ADH activity changes across timings were significant in roots but not in leaves (Figs 5 and 6). ADH activity in waterlogged roots of TF was greater than in the roots of RB (Fig 6). In roots of waterlogged TF, ADH activity increased 93, 45, 39, and 57% at 1, 2, 3, and 4 WOT. In roots of waterlogged RB, ADH activity increased 56, 27, 22, and 23% at 1, 2, 3, and 4 WOT.

**Fig 7:**
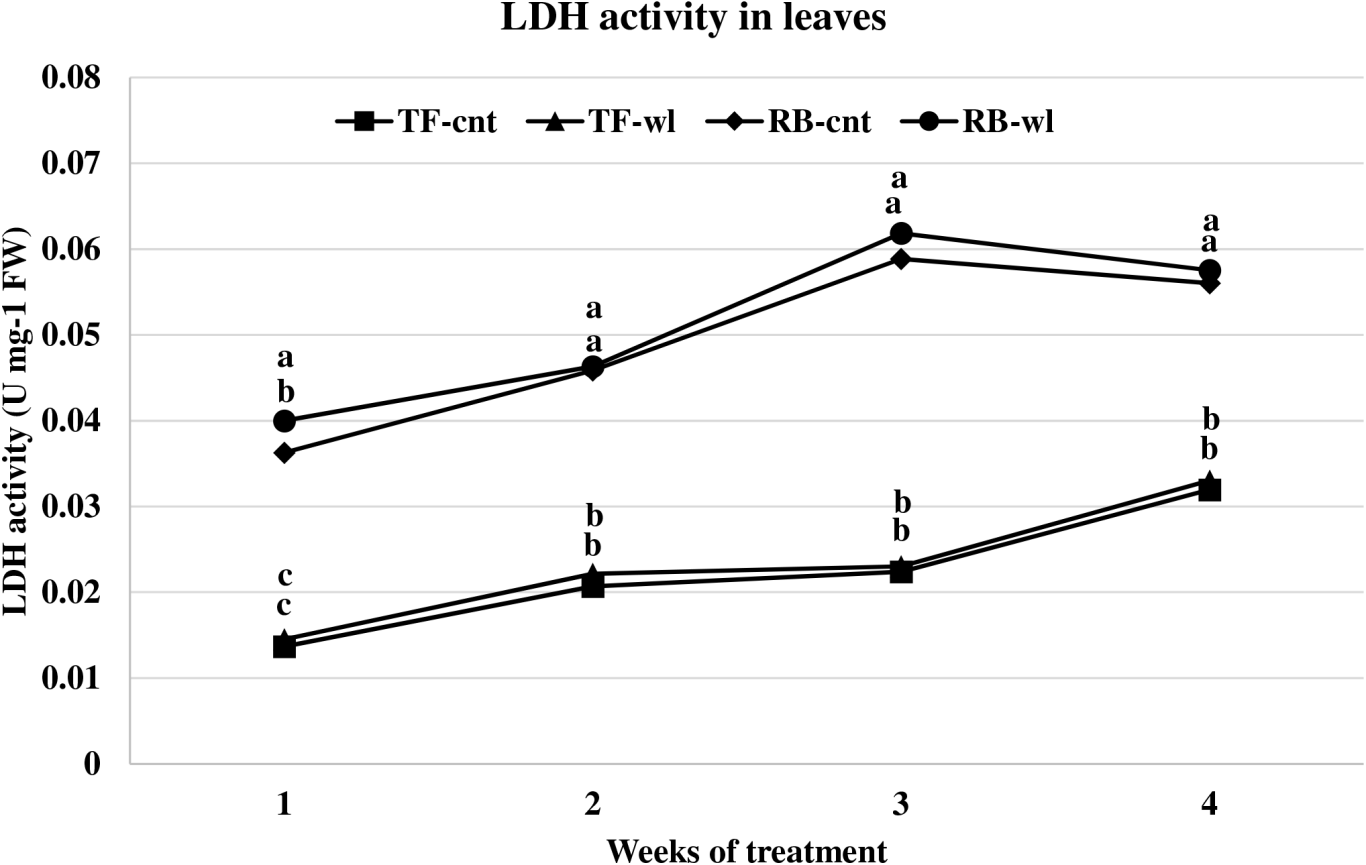
LDH activity changes in leaves of tall fescue (TF) and roughstalk bluegrass (RB). LDH activity changes in leaves of control tall fescue (TF-cnt), control roughstalk bluegrass (RB-cnt), waterlogged tall fescue (TF-wl), and waterlogged roughstalk bluegrass (RB-wl) during the 4-week greenhouse study. Data represent means of 32 individuals. Means in the same week with the same letter are not different based on Duncan’s multiple range test at 0.05 probability.

**Fig 8:**
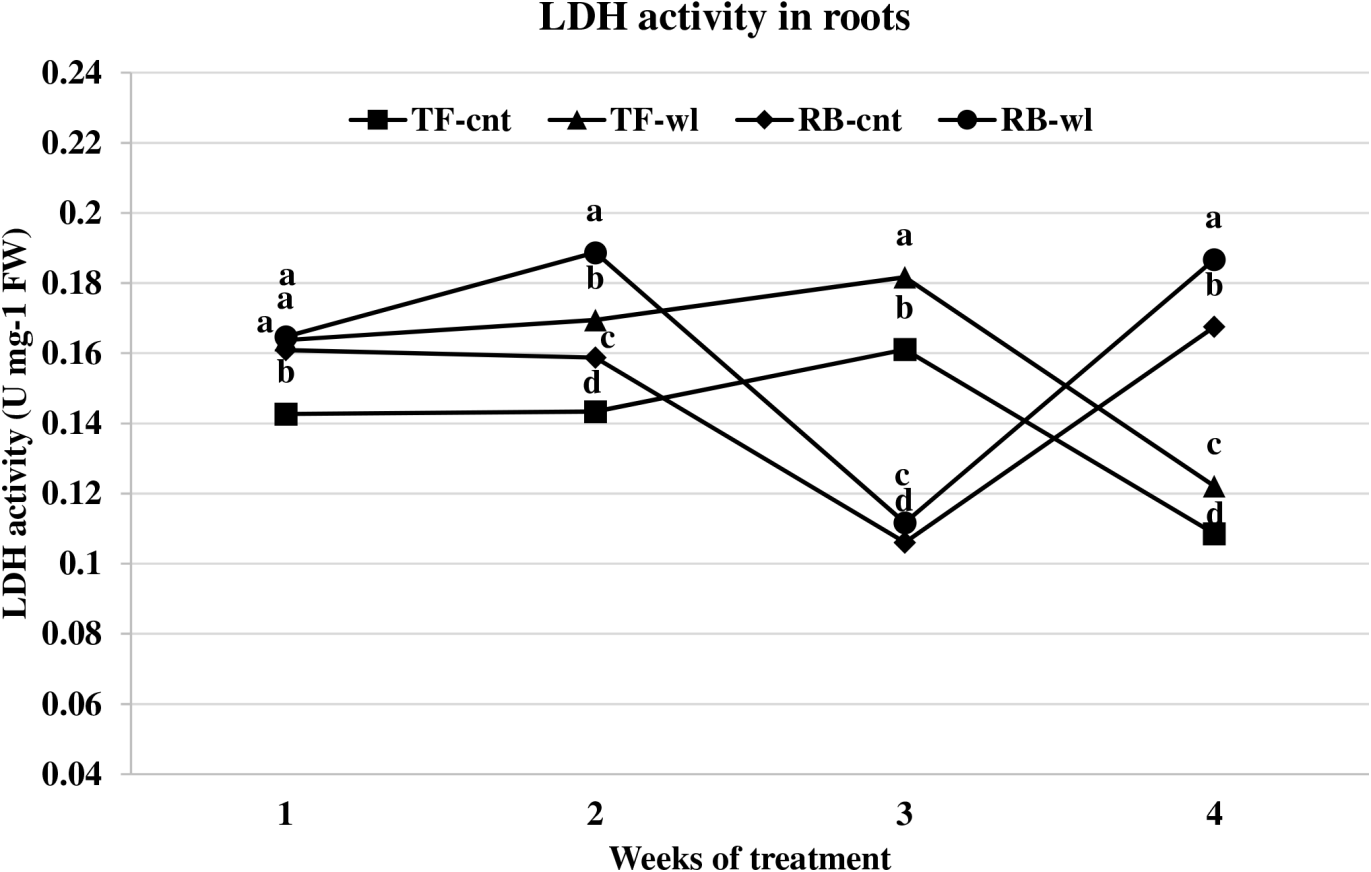
LDH activity changes in roots of tall fescue (TF) and roughstalk bluegrass (RB). LDH activity changes in roots of control tall fescue (TF-cnt), control roughstalk bluegrass (RB-cnt), waterlogged tall fescue (TF-wl), and waterlogged roughstalk bluegrass (RB-wl) during the 4-week greenhouse study. Data represent means of 32 individuals. Means in the same week with the same letter are not different based on Duncan’s multiple range test at 0.05 probability.

Changes of LDH activity in waterlogged TF and RB were similar to the changes for ADH activity. The LDH activities in leaves were not different between control and waterlogged plants in either TF or RB species during the 4-week study (Fig 7). In the roots of waterlogged plants, significant increases in LDH activity were observed in TF throughout the entire study period, and at 2, 3, and 4 weeks in RB. At 1, 2, 3, and 4 WOT, root LDH activity increased 15, 18, 13, and 13% in TF, and 2, 19, 5, and 11% in RB (Fig 8). The increases of root LDH activity were greater in TF than in RB across the 4-week study, except at 2 WOT.

The only leaf WSC concentration change under waterlogging conditions was observed in the leaves of RB plants at 2 WOT (Table 4). In contrast, waterlogging induced WSC concentration reductions were measured in the roots of both TF and RB. The root WSC concentration reductions were greater in TF (range 9.3 to 13.9%) than in RB (range 7 to 9%) across the 4-week study.

**Table 4:** Leaf water soluble carbohydrate (LWSC) content and root water soluble carbohydrate (RWSC) content in tall fescue (TF) and roughstalk bluegrass (RB) during the 4 week greenhouse study.

Population	Treatment^a^	LWSC (mg g^-1^ DW)^b^			
		Week 1	Week 2	Week 3	Week 4
TF	cnt	114.7aA	120.2aA	147.4bA	139.7bA
	wl	114.6aA	122.1bB	147.2cA	144.2cA
	Decrease (%)^c^	0.1	-1.6	0.1	-3.2
RB	cnt	144.1aA	136.5bA	159.6cA	163.0cA
	wl	143.0aA	131.6bB	164.3cA	159.8cA
	Decrease (%)	0.8	3.5	-2.9	1.9
		RWSC (mg g^-1^ DW)			
TF	cnt	104.8aA	101.2aA	107.7abA	114.7bA
	wl	90.2aB	91.9aB	97.7abB	103.2bB
	Decrease (%)	13.9	9.3	9.3	10.0
RB	cnt	96.3aA	98.4aA	108.7bA	108.0bA
	wl	89.7aB	89.8aB	99.4bB	100.2bB
	Decrease (%)	6.9	8.7	8.6	7.2

^a^ cnt = no treatment control; wl = waterlogging treatment.

^b^ data represent means of 32 individuals. Means followed by the same lower case letter in the row and upper case letter in the column are not different based on Duncan’s multiple range test at 0.05 probability.

^c^ percentage decrease in water soluble carbohydrate content for waterlogged plants, negative numbers represent an increase.

### Photosynthesis and chlorophyll measurements

**Fig 9:**
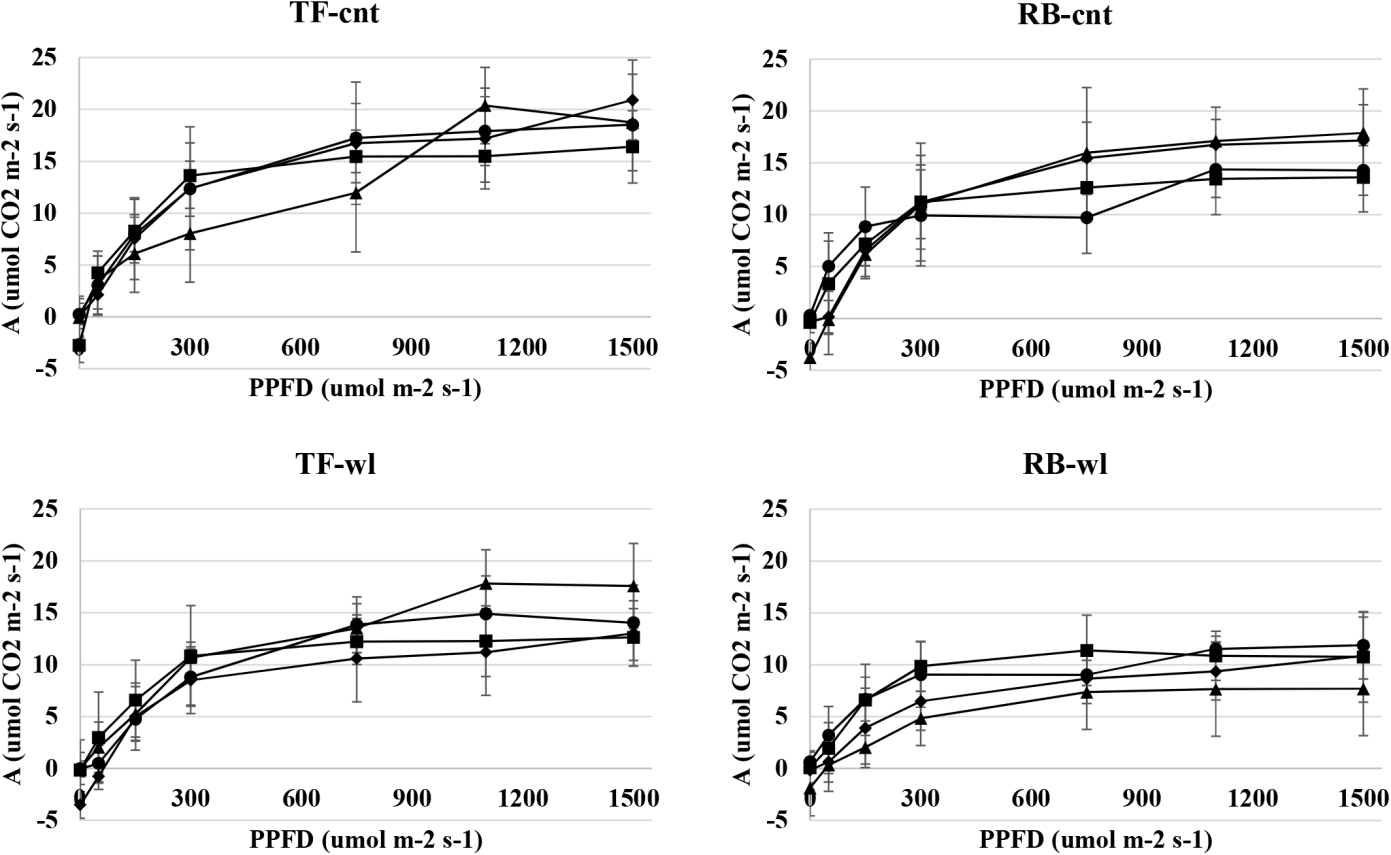
Response of leaf net photosynthetic rate in tall fescue (TF) and roughstalk bluegrass (RB). Response curves of leaf net photosynthetic rate (*A*) as a function of photosynthetic photon flux density (PPFD) in control tall fescue (TF-cnt), control roughstalk bluegrass (RB-cnt), waterlogged tall fescue (TF-wl), and waterlogged roughstalk bluegrass (RB-wl) at 1 (●), 2 (■), 3(▲), and 4 (♦) weeks of treatment. Data represent means of 8 individuals ±SE.

The maximum photosynthesis rates of TF and RB at 1500 umol m^-2^ s^-1^ (*A*_1500_) were reduced by the waterlogging treatment (P<0.05) (Fig 9). However, the reduction in photosynthetic rates was not different between species and was not related to the duration of waterlogging. The *A*_1500_ of control TF ranged from 16.40 to 20.91 umol CO_2_ m^-2^ s^-1^ during the four-week study with an average of 18.64 umol CO_2_ m^-2^ s^-1^. The average *A*_1500_ in waterlogged TF was reduced 23% and ranged from 13.01 to 17.57 umol CO_2_ m^-2^ s^-1^. *A*_1500_ of control RB ranged from 13.62 to 17.88 umol CO_2_ m^-2^ s^-1^ during the four-week study with an average of 15.73 umol CO_2_ m^-2^ s^-1^. The average *A*_1500_ in waterlogged RB was reduced 35%, and ranged from 7.70 to 11.87 umol CO_2_ m^-2^ s^-1^. The light compensation points were not different between the control and waterlogged plants for either TF or RB. Saturation points were slightly reduced in both waterlogged TF and RB. The saturation point for control TF and RB was near 500 mol m^-2^ s^-1^, and for waterlogged TF and RB was near 300 mol m^-2^ s^-1^.

**Fig 10:**
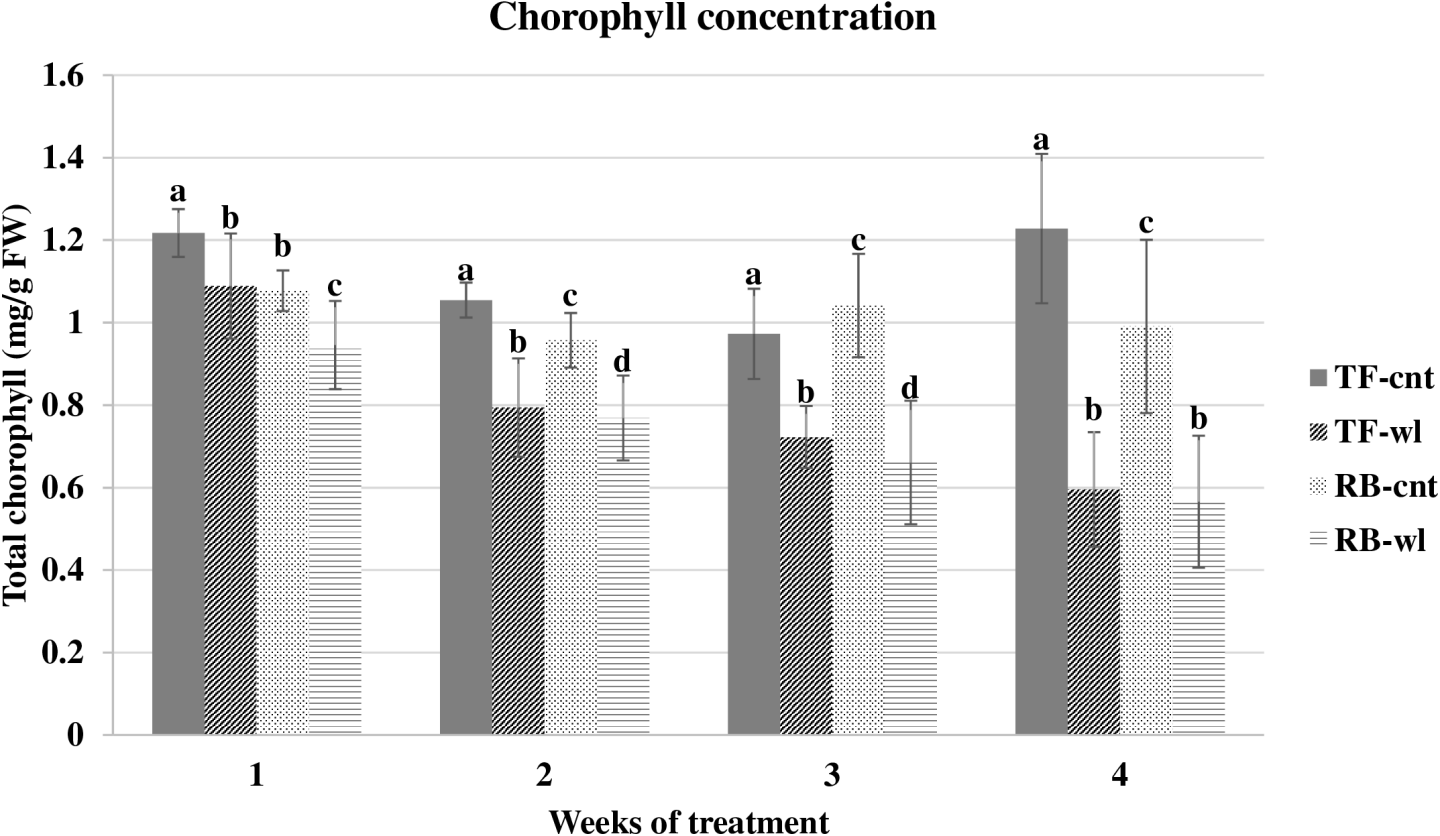
Chlorophyll concentration changes in tall fescue (TF) and roughstalk bluegrass (RB). Chlorophyll concentration in control tall fescue (TF-cnt), control roughstalk bluegrass (RB-cnt), waterlogged tall fescue (TF-wl), and waterlogged roughstalk bluegrass (RB-wl) during the 4-week greenhouse study. Data represent means of 32 individuals ±SE. Means in the same time period with same letter are not different based on Duncan’s multiple range test at 0.05 probability.

Significant decreases in chlorophyll concentration were observed in both waterlogged TF and RB compared to the controls (Fig 10). Chlorophyll concentration reduction increased with the duration of waterlogging (P<0.05). After 4 weeks of the waterlogging treatment, chlorophyll concentrations were reduced by 23 and 25% in TF and RB, respectively, but the reductions were not different between the two species.

## Discussion

Variation in morphometric and metabolic responses between TF and RB under waterlogging conditions indicated a potential differences in waterlogging tolerance between these two species. Differences in root development was the most distinguishable trait in response to waterlogging. Compared to TF, the distribution of the root system of RB was shallower in the waterlogged treatment. The shallower root system may help the RB roots to absorb more oxygen from the upper soil layers. The root diameter of RB was less than TF, and RB had more roots than TF. Root systems with this feature usually have a greater surface area to volume ratio [56]. This trait may improve waterlogging tolerance of RB, because root systems with larger surface area to volume ratio usually have greater nutrient absorption efficiency and gas diffusion rates [57, 58].

After four weeks of waterlogging, aerenchyma increased in waterlogged RB and TF. The increase of aerenchyma in waterlogged RB was greater than in waterlogged TF, which may contribute to better oxygen transport and to a lower oxygen consumption rate in waterlogged RB. Lysigenous root aerenchyma does not typically form in well-drained dryland species such as maize (*Zea mays*), wheat (*Triticum aestivum*), and barley (*Hordeum vulgare*), but may be induced by poor aeration [12, 59]. Root aerenchyma formation was observed in untreated TF and RB, which may indicate that TF and RB have a similar, but low oxygen-stress threshold. The oxygen deficiency level could influence the formation rate and area of root aerenchyma. Although the formation of aerenchyma in dryland species is less extensive than in wetland species [13], aerenchyma formation in dryland species usually occurs within several hours of perception of oxygen deficiency [60, 61]. Interestingly, the waterlogged RB did not produce a greater proportion of root aerenchyma and porosity until 2 WOT. A possible explanation is that the RB has a lower oxygen requirement. Because the development of waterlogging induced oxygen deficiency is a slow process, RB may not reach the low oxygen stress threshold at 1 WOT. This feature may help delay aerenchyma formation during short-term waterlogging, and minimize the loss of root function resulting from cell death during aerenchyma formation.

Adaptive root metabolic response is one of the most important factors that contributes to plant waterlogging tolerance [16, 62]. In this study, root metabolism was more sensitive to waterlogging than leaf metabolism in both TF and RB. Changes in alcohol and lactic acid fermentation usually represent adaptive strategies for waterlogging tolerance [62]. Increased ADH and LDH rates indicated oxygen deficiency occurred in waterlogged roots of both TF and RB. The lower ADH activities in waterlogged roots of RB plants indicated a lower fermentation rate compared with waterlogged TF plants. The mechanism for the lower fermentation rate was not evaluated in this study. However, it may be due to better oxygen diffusion as a result of the shallower root system or because of lower metabolism under stress. Under the same conditions, lower fermentation activity produces less toxic metabolites and causes less damage to the plant cells [16, 18]. Thus, RB plants may have less waterlogging induced cellular damage than TF plants. Depletion of respirable substrates may occur in roots, because sugars are not delivered to the apical zone during waterlogging [16]. Though sugar concentrations are high at the whole plant level, sugar transport in phloem can be reduced in waterlogged roots due to reduced energy production [63]. For example, increased leaf WSC concentration and decreased root WSC were observed in waterlogged Kentucky bluegrass plants [38]. However, based on the results of this study, the root respiration of these two species was not influenced by lack of fermentation substrate. The root WSC concentrations were reduced in both waterlogged RB and TF plants, but the reductions were not correlated with the duration of waterlogging.

In TF and RB, the maximum photosynthetic rates were reduced by waterlogging. Leaf chlorophyll content decreased in waterlogged TF and RB plants over the four treatment times. Chlorophyll is one of the most important photosynthetic pigments in higher plants, and chlorophyll content is often influenced by environmental stress [64, 65, 66]. Thus, the reduction of chlorophyll content in waterlogged TF and RB plants may explain the reduction in photosynthetic capacity in these plants. Furthermore, according to the light response curves, the effective quantum yields were slightly less in waterlogged TF and RB plants. Because the effective quantum yield is proportional to efficiency of photosynthetic pigments for light capture [67, 68], these changes give supporting evidence that the photosynthetic pigments were influenced by waterlogging. Some morphological responses, such as plant height and by leaf numbers, were different between RB and TF. Because photosynthesis is mainly influenced leaf function [69, 70, 71], less reduction in leaf number and plant height may help waterlogged RB maintain photosynthetic yield compared to TF.

## Conclusions

Compared with TF, several adaptive characteristics observed in this study may contribute to better waterlogging tolerance in RB. The shallower root system and larger aerenchyma areas may contribute to better oxygen use efficiency. Lower fermentation rates produce less toxic anaerobic metabolites and minimize waterlogging induced damage. The results of this study improved our understanding of RB survival in cool season grass seed production fields, and the adaptations of this species to low-oxygen stress caused by waterlogging. However, plants of both TF and RB survived the four week long simulated waterlogging treatment suggesting that research should be expanded to other waterlogging induced stress responses such as osmotic stress and salt toxicity, soil nutrient leaching or hyperoxia (post-waterlogging), and should be conducted at different or more plant growth stages.

## Supporting information

S1 Fig ADH activity changes in leaves of tall fescue (TF) and roughstalk bluegrass (RB). ADH activity changes in leaves of control tall fescue (TF-cnt), control roughstalk bluegrass (RB-cnt), waterlogged tall fescue (TF-wl), and waterlogged roughstalk bluegrass (RB-wl) during the 4-week greenhouse study. Data represent means of 32 individuals. Means in the same week with the same letter are not different based on Duncan’s multiple range test at 0.05 probability.
(XLSX)

S2 Fig ADH activity changes in roots of tall fescue (TF) and roughstalk bluegrass (RB). ADH activity changes in roots of control tall fescue (TF-cnt), control roughstalk bluegrass (RB-cnt), waterlogged tall fescue (TF-wl), and waterlogged roughstalk bluegrass (RB-wl) during the 4-week greenhouse study. Data represent means of 32 individuals. Means in the same week with the same letter are not different based on Duncan’s multiple range test at 0.05 probability.
(XLSX)

S3 Fig LDH activity changes in leaves of tall fescue (TF) and roughstalk bluegrass (RB). LDH activity changes in leaves of control tall fescue (TF-cnt), control roughstalk bluegrass (RB-cnt), waterlogged tall fescue (TF-wl), and waterlogged roughstalk bluegrass (RB-wl) during the 4-week greenhouse study. Data represent means of 32 individuals. Means in the same week with the same letter are not different based on Duncan’s multiple range test at 0.05 probability.
(XLSX)

S4 Fig LDH activity changes in roots of tall fescue (TF) and roughstalk bluegrass (RB). LDH activity changes in roots of control tall fescue (TF-cnt), control roughstalk bluegrass (RB-cnt), waterlogged tall fescue (TF-wl), and waterlogged roughstalk bluegrass (RB-wl) during the 4-week greenhouse study. Data represent means of 32 individuals. Means in the same week with the same letter are not different based on Duncan’s multiple range test at 0.05 probability.
(XLSX)

S5 Fig Response of leaf net photosynthetic rate in tall fescue (TF) and roughstalk bluegrass (RB). Response curves of leaf net photosynthetic rate (*A*) as a function of photosynthetic photon flux density (PPFD) in control tall fescue (TF-cnt), control roughstalk bluegrass (RB-cnt), waterlogged tall fescue (TF-wl), and waterlogged roughstalk bluegrass (RB-wl) at 1 (●), 2 (■), 3(▲), and 4 (♦) weeks of treatment. Data represent means of 8 individuals ±SE.
(XLSX)

S6 Fig Chlorophyll concentration changes in tall fescue (TF) and roughstalk bluegrass (RB). Chlorophyll concentration in control tall fescue (TF-cnt), control roughstalk bluegrass (RB-cnt), waterlogged tall fescue (TF-wl), and waterlogged roughstalk bluegrass (RB-wl) during the 4-week greenhouse study. Data represent means of 32 individuals ±SE. Means in the same time period with same letter are not different based on Duncan’s multiple range test at 0.05 probability.
(XLSX)

S1 Table Turf quality, leaf number, height, aboveground dry biomass, root dry biomass, root length, and root distribution of tall fescue (TF) and roughstalk bluegrass (RB) under control and waterlogging conditions. (XLSX)

S2 Table Changes in aerenchyma area in the root cross sections of tall fescue (TF) and roughstalk bluegrass (RB) during the 4 week greenhouse study. (XLSX)

S3 Table Leaf water soluble carbohydrate (LWSC) content and root water soluble carbohydrate (RWSC) content in tall fescue (TF) and roughstalk bluegrass (RB) during the 4 week greenhouse study. (XLSX)
